# Protected learning time in community pharmacy and possibilities for upscaling: an exploratory study in Wales, UK

**DOI:** 10.1080/20523211.2026.2629063

**Published:** 2026-02-20

**Authors:** Sophie Bartlett, Alison Bullock

**Affiliations:** Cardiff Unit for Research and Evaluation in Medical and Dental Education, School of Social Science, Cardiff University, Cardiff, UK

**Keywords:** Continuing professional development, protected learning time, education and training, pharmacist development

## Abstract

**Background:**

The remit of pharmacists’ vital role in the healthcare system is expanding, and continuous professional development (CPD) of pharmacists is critical. While CPD is mandatory in the UK, a lack of protected learning time (PLT) hinders engagement, particularly in community pharmacies. In Wales, UK, a national PLT programme was piloted to address this, involving funding for 12–15 days of PLT for community pharmacists. This study investigated whether PLT provision can benefit both community pharmacists and their pharmacy, and explored opportunities for upscaling PLT provision.

**Method:**

A realist qualitative approach was adopted across four phases. Community pharmacists participating in the PLT programme submitted monthly diary entries and engaged in one-off group and individual interviews. Employer perspectives were collected via an online survey. Education and Training Leads reflected on preliminary findings and their comments were captured via group interview. Data were coded and analysed thematically through a constant comparative approach.

**Results:**

Thirty participants contributed data, including 96 diary entries from 20 pharmacists, interviews with 15 pharmacists, survey responses from 12 employers, and input from 3 education leads. The PLT promoted both individual professional development and organisational capacity, and also enhanced pharmacists’ personal wellbeing. Nonetheless, challenges for employers were prevalent, including rising costs of locum cover and service disruptions. Participants proposed two scalable PLT models: pre-scheduled PLT slots and non-patient-facing hours.

**Conclusion:**

PLT improves pharmacist professional development and personal wellbeing and enables broader service provision in their pharmacy. However, upscaling of PLT requires addressing financial and logistical barriers. Structured and equitable PLT models, such as routine closures or non-patient-facing hours, warrant further research and piloting to assess feasibility, acceptability and impact both for pharmacists and patient care.

## Background

The human population is growing and living longer. Medication is becoming progressively more complex, and the prevalence of polypharmacy continues to rise (Guthrie et al., 2015). As experts of medicines, it is no surprise that pharmacists’ roles are expanding and becoming more critical to addressing pressures on healthcare services.

As healthcare demands evolve, pharmacists face ongoing changes in legislation, medicine guidelines and drug therapies. Historically, pharmacists have been responsible for the correct supply of medications, however, today they are expected to provide clinical advice, consultations, manage chronic conditions and even prescribe medication (John, 2018; Young & Anderson, 2024). In early 2021, the General Pharmaceutical Council (GPhC) in the UK published new standards for the initial education and training of pharmacists. Such standards were updated in order to transform the pharmacy workforce and meet the changing healthcare needs of the UK population (General Pharmaceutical Council, 2025). A key development in the standards is that, as of the first day of practicing as a qualified and registered pharmacist, pharmacists will have independent prescriber (IP) status. This means that pharmacists will be able to prescribe autonomously for certain conditions within their scope of practice. However, as this is a revision to initial education and training, it threatens a mismatch between the incoming pharmacist workforce and the legacy workforce, who completed their training prior, and will not necessarily have prescriber status.

Continuing professional development (CPD) is therefore more paramount than ever to ensure that existing pharmacists keep pace with their evolving role and remain up-to-date with medicine guidelines and processes for providing safe patient care. CPD is mandatory for pharmacists across many international jurisdictions. For UK pharmacists, while CPD has been a requirement since 2005 (Attewell et al., 2009), it is only since 2022 that UK pharmacists are required to submit evidence of four CPD activities, one peer discussion, and one reflective account each year to maintain their registration (Pharmacy Magazine, 2022).

Research into pharmacists’ engagement with and experiences of CPD is a global endeavour, and common trends are evident among the findings. The importance of CPD is increasingly recognised and supported by pharmacists, however, common barriers such as lack of time, remuneration, and staff cover are prevalent internationally (Attewell et al., 2009; Clifford et al., 2011; Donyai et al., 2011; Saade et al., 2018; Swallow et al., 2006; Wilbur, 2010). One multi-country study found that pharmacists commonly relied on their personal time to engage in learning and development (Micallef & Kayyali, 2022).

In the UK, the Royal Pharmaceutical Society (RPS) advocates that all pharmacists should have access to Protected Learning Time (PLT) within their work hours to pursue education and training (Royal Pharmaceutical Society, 2023a). Despite this, PLT is not routinely provided (Willis, 2025). The National Health Service (NHS) contract for community pharmacists does not mandate PLT, therefore placing provision at the discretion of the employers. Historically, hospital and primary care pharmacists in the UK have devoted more time to CPD than their community-based colleagues (Power et al., 2007; Swainson & Silcock, 2004). This pattern is echoed internationally. In both New Zealand and the USA, hospital pharmacists report access to PLT where community pharmacists typically do not (Micallef & Kayyali, 2022).

Time pressures remain a particularly acute barrier for community pharmacists. Recent studies in the UK and Jordan highlight this challenge, with community pharmacists identifying insufficient time as a key barrier to CPD participation and upskilling (Qaisi et al., 2025; Wilkinson, 2024). Supporting this, a UK RPS workforce survey on wellbeing demonstrated that 93% of community pharmacists were provided with none or insufficient PLT, compared to 61% across all sectors (Royal Pharmaceutical Society, 2023b). Pharmacists have also reported that the absence of PLT has a negative impact on their mental health and risk of burnout (Royal Pharmaceutical Society, 2023b; Smail, 2023).

There is an apparent disconnect between the importance and desire for CPD and the accessibility and opportunity for engagement. However, PLT comes with a financial cost. In community pharmacies in particular, there may only be one pharmacist on site at a time, and the release of this pharmacist therefore requires backfill. In an attempt to make CPD more accessible to community pharmacists, Health Education and Improvement Wales (HEIW), the strategic workforce body for NHS Wales working to address strategic and specialist workforce issues, initiated a pilot programme that offered funding to community pharmacies to cover 12–15 days of PLT during work hours over a 10-month period to support the development of pharmacists. Any community pharmacists could apply to the programme on the condition they were not already engaging in some form of funded CPD scheme. Development could take a number of different forms: pursuing credit-bearing qualifications through university courses, or self-directed learning against a recognised framework. These could include advanced practice, or independent prescribing status permitting pharmacists to prescribe autonomously for certain conditions. Pharmacists could also use the PLT to cover their own time and that of a dedicated mentor who can observe, assess competencies and provide feedback. Pharmacists had to apply, but funding was routed through their employer and had to be used between October 2021 and July 2022. The programme was open to all community pharmacists across Wales (approximately 1,000 as of 2019 [Health Education and Improvement Wales, 2019]).

Although existing literature highlights pharmacists’ desire for PLT and the barrier its nonavailability presents to accessing CPD, there is a lack of evidence to demonstrate (a) whether provision of PLT yields benefits to both community pharmacists and their pharmacy, and (b) if there are benefits, whether there is scope to offer PLT on a larger scale. The aim of this study was to explore these two questions, in the context of the PLT programme in Wales.

## Methods

### Study design

The study employed a four-phase design to explore the experiences and perspectives of pharmacists participating in the PLT programme, as well as their employers and Education and Training (E&T) Leads. A realist qualitative approach was adopted to understand how, for whom, and under what circumstances the PLT programme was experienced as effective. This approach assumes that participants’ accounts reflect an underlying reality, shaped by both individual and contextual factors (Pawson & Tilley, 1997).

As the PLT programme targeted the existing workforce, it welcomed pharmacists with varying degrees of experience and expertise who sought to use the time for different purposes. Given such complexity, a realist approach enabled the research team to explore how different stakeholders experienced and made sense of the programme. It supported the identification of nuances across individual pharmacists as well as key mechanisms (e.g. time protection, support structures) and how these interacted with contextual conditions to influence outcomes. The sequential phases of data collection were designed to collect in-depth, multi-stakeholder qualitative data through diaries, free-text questions on surveys, interviews, and discussions with E&T leads.

### Study setting and participants

HEIW acted as the gatekeepers for this study. All pharmacists enrolled in the PLT programme and their employers were invited to participate. Additionally, E&T Leads in HEIW’s pharmacy division participated in the fourth and final phase of the study.

### Data collection procedures

All data collection instruments (see Supplemental Material) were developed by the research team based on their own previous research experience in pharmacy settings, results and limitations of relevant literature described previously, and the key components of the PLT programme. Instruments were validated via review from and discussion with HEIW staff, including active community pharmacists and education and training leads.

The four data collection phases occurred between October 2021 and September 2022:

- Phase 1 – Pharmacists’ Monthly Online Diaries (October 2021–July 2022): Pharmacists on the programme were sent monthly invitations via email to complete an online diary entry reflecting on their use of PLT during that month and any benefits experienced.
- Phase 2 – Pharmacist Interviews (March–April 2022): Pharmacists were invited via HEIW to attend an online group interview at selected dates conducted via Microsoft Teams. Those interested contacted the research team directly and were provided with further details of the study and a consent form. Pharmacists unable to attend group interviews were offered a one-to-one telephone or online interview. Group interviews were conducted by either author SB or AB; all one-to-one interviews were conducted by SB. Interviews were recorded and transcribed verbatim.
- Phase 3 – Employer Survey (June–July 2022): HEIW distributed an online survey to employers of pharmacists on the programme on behalf of the researchers. Based on HEIW’s advice regarding employers’ time constraints, a survey was selected over interviews. To support qualitative data collection, the survey largely comprised open-text response questions.
- Phase 4 – E&T Leads Discussion (September 2022): Preliminary findings from Phases 1–3 were shared with HEIW E&T Leads via teleconference. The meeting included a structured group discussion to elicit their reflections and commentary, which were also analysed as part of the study.

### Analytical approach

While some quantitative data were captured in online diaries and categorical data from employers’ surveys, data were primarily qualitative. Addressing the research questions required a focus on participants’ perspectives and their accounts, as such, we report only qualitative data here. All qualitative data, including diary entries, interview transcripts and open-text survey responses, were imported into NVivo software (version 12) for coding and analysis.

Data from Phases 1–3 were analysed concurrently to allow early insights to inform the discussion in Phase 4. This involved sharing and discussing the emerging themes with E&T Leads, who were invited to provide commentary and feedback on the authors’ interpretations and share their reflections on the value of and place for the PLT programme.

A thematic analysis was conducted inductively using a constant comparative method (Glaser, 1965). Data were coded as either a context, mechanism, or outcome in line with a realist approach (Pawson & Tilley, 1997). Data coded as ‘context’ described elements outside of the PLT programme itself but were directly influential to subsequent outcomes (Greenhalgh & Manzano, 2022), ‘mechanisms’ were data describing underlying processes that led to particular outcomes (Greenhalgh et al., 2015), and ‘outcome’ was coded to any data describing direct results of the PLT programme. Initial coding was conducted by author SB, with all codes reviewed by author AB. Coding decisions and emerging themes were agreed upon through joint discussion.

As each new data item was coded, it was compared to previously coded items to identify its place as a context, mechanism or outcome. This supported robust theme development across participants and stakeholder groups. This approach was chosen to support cross-case and cross-group comparisons, making it well suited to understanding the experiences of pharmacists, employers, and educational leads (Boeije, 2002). It also complements the sequential analysis of data from phases 1–3 and then later 4 by permitting a cycle of comparison and reflection on ‘old’ and ‘new’ data (Boeije, 2002). Data saturation was reached during the analysis process.

### Ethical procedures

Information on the study and invitations (see Supplemental Material) was prepared by the researchers and distributed to potential participants by HEIW on their behalf. Ethical approval was granted by the Research Ethics Committee within the School of Social Sciences at Cardiff University (committee reference #33). All participants provided opt-in consent prior to participation: pharmacists through diary and interview consent forms, employers through survey consent, and E&T Leads through a written agreement. All data were anonymised, and participants are referred to by role only (e.g. pharmacist, employer, E&T Lead).

## Results

The PLT programme received 42 applications. Four applications were declined by HEIW either because applicants were already receiving some level of funding for training and development, or their application did not provide sufficient detail on how they intended to use the PLT. Six pharmacists dropped out of the programme. In total, 32 community pharmacists completed the pilot programme. All 32 were invited to participate in phases 1 and 2, and 24 participated either through diary entries (phase 1), interviews (phase 2) or both. Twenty of these 24 pharmacists submitted online diary entries across 10 months, totalling 96 entries and 7,578 words of text. Pharmacists each submitted between two and nine monthly entries. Eleven of the 24 pharmacist participants participated in one of three group interviews and four participated in a personal interview (15 pharmacists in total). All participants in group interviews contributed to discussions. In terms of pharmacists, data saturation was deemed to be reached after the group interviews. Although subsequent participants continued to provide further examples and contexts of their experiences, no new mechanisms or outcomes emerged beyond those already identified. This indicated that the data had sufficient depth and breadth to address the research question. Nonetheless, all interviews were used in analysis. Twelve employers participated via their responses to the online survey, nine of whom were completing the PLT programme themselves and so also participated as pharmacists. The three E&T Leads at HEIW involved in the oversight of the programme implementation participated in a group interview. In total, data were collected from 30 participants.

A total of four hours and 15 min of conversation data was obtained through conversations with pharmacists and E&T Leads. Group interview durations ranged from 34 to 45 min (four group interviews, 40 min average) and personal interviews ranged from 20 to 30 min (four personal interviews, 24 min average).

In the following sections, results are presented according to key context-mechanism-outcome (CMOs) configurations identified through data analysis. We begin by outlining the immediate outcomes of the PLT programme, which are grouped into two overarching themes: the benefits of the PLT programme, and its impact on employers. We then explore future directions for PLT, focusing first on the sector-specific context of community pharmacy, and subsequently on the potential mechanisms for upscaling PLT.

All data sources were analysed collectively and are reported together to provide a more integrated and contextually rich account of the CMO configurations. This approach offers a more holistic and coherent understanding of the dynamics within community pharmacy than would be possible by reporting findings by participant group or data collection method in isolation. A visualisation of the CMO configurations is presented in Table 1, and these are aligned with the two research questions in the discussion section.

**Table 1.** Context, mechanisms and outcomes from data.

		**Context**	**Mechanism**	**Outcome**
**Now (PLT programme)**	Outcome: Benefits of PLT	Locum availability Isolation of community Time available for training Demand for PLT Arranging time Company challenges	Support from employers Funding Reflection Release from service provision Manage workload Protected personal time Highlights importance of CPD	Day-to-day practice Expansion of scope Patient care / service provision Achievements / qualifications Confidence Support from others Release from service provision Working with other healthcare professionals
	Outcome: Impact on Employers	Insufficient funding Locum availability Arranging time Company challenges New Initial Education and Training standards for pharmacists	Like-for-like backfill Financial Productivity	Day-to-day practice Expansion of scope Patient care / service provision
**Future (upscaling)**	Sector-specific Contexts in Community	Isolation of community Comparison with General Practice settings Comparison with hospital settings Time available for training		
	Mechanisms for upscaling	Demand for PLT Lessons from Covid-19 New Initial Education and Training standards for pharmacists Locum availability Comparison with GP settings Time available for training Funding	Patient-facing hours Routine closures Top-down organisation Support from employers Nationally protected time	

### Outcome: benefits of protected learning time

Two key benefits of PLT were apparent from pharmacists’ diary entries. One was the opportunity to upskill and provide additional services, particularly in independent prescribing (IP). This was seen not only as advantageous to the individual pharmacist, but to their employer and their patients as it expanded the offer of services within the pharmacy:

It has enabled me to quickly get both the confidence and competence to rapidly upskill in my IP. This has allowed me to provide a service that is massively beneficial to the pharmacy, the local practices and the patients. (Pharmacist, Diary Entry)

This multifaceted benefit was confirmed in survey responses from employers who highlighted benefits of PLT provision such as ‘increased scope of practice to meet demand’ and ‘increased service delivery and patient satisfaction’.

The second key benefit was the opportunity to be released from service to undertake CPD, providing an ‘*uninterrupted space to learn’* (Pharmacist, Diary Entry) without the need to sacrifice personal time. This was particularly important to those using the time to pursue structured courses or qualifications with strict deadlines:

The protected time is invaluable as it allows us the extra time and support to juggle academic hours on top of day-to-day duties as a homeowner and family person. (Pharmacist, Diary Entry)

Some pharmacists reflected a belief that if they had undertaken CPD in their personal time, the additional workload burden would have jeopardised their performance and risked successful completion of their course:

The work pressure is immense and this time allows us to focus on study. I do not think I would have passed this far had it not been for the protected time. (Pharmacist, Diary Entry)

Employers, in turn, recognised how PLT did not just provide professional benefits, but personal ones too, prompting positive effects on morale and wellbeing. They highlighted how PLT had ‘improved that staff member’s morale’ and how ‘employee wellbeing was being addressed’.

### Outcome: impact of PLT on employers

Employers responded to the PLT programme with mixed views. Some appeared to have been more supportive of development opportunities since the introduction of the new UK pharmacy standards, which emphasise the importance of having prescribing pharmacists, and an increased access to funding:

They [employer] have had a recent switch in attitude towards prescribing courses, I think because one, the funding has been improved and two, with the way the new contract is made up, it’s very focused on having independent prescribers in the branches. So, it’s beneficial to them. They have been very supportive. (Pharmacist, Interview)

Others, however, while they recognised the long-term value, were reluctant to support the PLT programme due to immediate operational challenges, particularly in sourcing backfill:

Looking from the employer point of view, ultimately, there is no doubt that they will benefit from this, from our qualifications. But looking at the short-term balance, it probably was a struggle for them to release us to do the training because of the circumstances currently on the on the local market. (Pharmacist, Interview)

Locum shortages and rising fees were common concerns. If pharmacies do not have available staff in their teams to fill rota gaps, then they must source locums; self-employed pharmacists who work on a temporary basis, but who can charge higher hourly rates than a contracted pharmacist:

My employers weren't as keen to release me for the time as they felt that the backfill payments they were going to be receiving weren’t sufficient to cover the time. (Pharmacist, Interview)

Survey responses from employers provided their first-hand perspectives. Eleven of the 12 employers reported challenges in sourcing backfill. Some highlighted the challenge not only in sourcing a locum but also in sourcing one with the equivalent skillset and expertise of the pharmacist they were releasing. This led to reduced productivity or even the cancellation of some clinics. In some instances, this also presented a financial loss to the pharmacy as the NHS provides payment to community pharmacies when they offer advanced and enhanced services:

Locums are never as knowledgeable and productive as the staff member that is away on protected development time. (Employer, Survey Response)

The employee is an independent prescriber [IP]. It was difficult to obtain a Locum IP so service provision was slightly affected. […] As an employer, I would not want to lose out on our monthly IP payments due to PLT. (Employer, Survey Response)

### Context: sector-specific pressures in community pharmacy

Participants noted in interviews that community pharmacists face unique business pressures compared to peers in hospital and general practice settings. The commercial nature of community pharmacy was seen to bring greater financial pressures, leaving less flexibility for PLT:

Having worked with GPs … they all had much more time on their hands. […] It is a different pressure on the time of community pharmacists, because they work in a business environment where the pressure's on to deliver the business, pay the bills, pay the staff… (Pharmacist, Interview)

E&T Leads remarked on how community pharmacists often operate as sole practitioners, raising concerns about professional isolation and greater risk to patient safety and quality of care. They saw PLT as a necessary mechanism to connect these pharmacists with other healthcare professionals as part of multidisciplinary teams to foster collaborative learning and service provision:

Somebody who's a sole practitioner, they are more risky. It is not their lack of professionalism, it's just they don't have that professional interaction. If we want community pharmacists to be embedded in a multidisciplinary team, the only way that's going to happen is for people to have time to be able to have those discussions. (E&T Lead, Interview)

There were also concerns about workforce retention. Pharmacists worried that lack of PLT would drive colleagues to hospital and general practice settings where PLT was seen to be more readily available:

We know that our primary care and secondary care colleagues are having protected time. We need to make sure that community pharmacy is not seen as a disadvantage. (Pharmacist, Interview)

We have to get protected time for the colleagues. Otherwise, we’ll lose them, the best ones will leave. (Pharmacist, Interview)

### Mechanisms: opportunities for upscaling PLT

Participants endorsed the upscaling of PLT, expressing a unanimous view that PLT was invaluable to pharmacists’ development. They also felt the need for PLT was progressively rising as community pharmacists face increasing pressures to upskill in line with their expanding role:

It's got to be a recommendation that we provide pharmacy teams nationally protected time for development because of all these new services they have to do. (E&T Lead, Interview)

Nonetheless, participants caveated their support with their recognition of the logistical challenges of wider implementation, particularly around staff cover and associated financial burden. Two distinct models of PLT were identified among discussions. One involved regular pre-determined time slots for PLT to facilitate advanced planning and arrangement of staff cover:

If it was structured, for instance, one day per month, then that makes it quite feasible if it's on a rolling basis to employ another pharmacist to backfill on a regular routine for those absences. (Pharmacist, Interview)

Pharmacists suggested that if this were orchestrated at a higher organisation level, time slots could be arranged to ensure minimal impact on patient services. One suggestion was that community pharmacies within a geographical radius could take their PLT on different days to ensure appropriate backfill and alternative options for patients:

It doesn't have to be exactly the same day, let's say if it was a Monday for this branch, Tuesdays for the other branch … so it's easily predictable and easy to get the locums or colleagues to cover that time. (Pharmacist, Interview)

Others, although welcomed the regularity, remained concerned that the locum crisis could worsen in the future. They argued that a safer approach to upscaling PLT would be one that did not rely on sourcing cover from pharmacists. The second model posed with this cerebration was the introduction of non-patient-facing hours in community pharmacies. This would remove the requirement for any financial exchange and was perceived by some as a more reliable, sustainable, and equitable approach. Participants suggested this could be orchestrated by the Health Boards. In Wales, there are seven geographical Health Boards that are responsible for the organisation and delivery of care services in their area:

If the Health Board agreed that on a Wednesday afternoon with six weeks notice, all pharmacies in that cluster were to close and come together to do an educational session. That would not mean that there would be any financial burden to HEIW, to the Health Board, to the pharmacies, and no one’s disadvantaged because it’ll be the whole cluster. (Pharmacist, Interview)

Some felt that changes to services during the Covid-19 pandemic served as evidence to support such a model. During this time, it was commonplace for community pharmacies in the UK to close for short periods to manage the increased pressures and workload. This demonstrated short closures as a viable option, not detrimental to patient services:

Community pharmacy doesn't need to be providing patient facing services for 45 hours a week, It’s OK to close for half a day. I think that we’ve probably got enough data to show that during Covid, a lot of pharmacies did close for two hours during the daytime. And as far as I'm aware, there was no adverse event that occurred in that time. (Pharmacist, Interview)

Nonetheless, the proposition of regular pharmacy closures prompted debate among participants. Some expressed reservations and felt the walk-in service nature of community pharmacy meant they should remain ‘*open and accessible to patients*’ (Pharmacist, Interview):

In GP surgeries it works because all the appointments are booked in advance, and can be moved around, while pharmacy is more demand-and-supply, and so closing, it’s not necessarily the best option here. (Pharmacist, Interview)

One E&T Lead underlined the caveat that being open and accessible is only of benefit if the pharmacy can offer the required services. They stressed a case of a ‘catch-22’ whereby the very need for PLT is pressed by the expanding role of community pharmacists and their increasing services, but the absence of time to develop and train in these areas inhibits the provision of such services. They argued for a pragmatic approach to ensure professional development whilst maintaining patient services:

If people can't develop themselves, they can't provide the service anyhow. So, at some point, you’ve got to have a pragmatic view of how do we support these people to get the development they need to provide the best patient services? (E&T Lead, Interview)

## Discussion

This study examined the implementation and outcomes of PLT within community pharmacy and explored potential mechanisms for upscaling its provision. The findings demonstrate how PLT promotes both individual professional development and organisational capacity. Pharmacists were able to expand their scope of practice, such as through independent prescribing, which subsequently broadened the services their pharmacy could offer. Also important is the finding that the PLT enhanced pharmacists’ personal wellbeing, through the mechanism of safeguarded time for development without encroaching on personal life. This is particularly salient in the context of recent workforce data showing that community pharmacists are more likely to report poor mental health than colleagues in other sectors (Royal Pharmaceutical Society, 2023b).

The international literature consistently recognises both the importance of CPD and the barriers that limit pharmacists’ access to it, with a lack of time being identified as a particularly acute challenge (Attewell et al., 2009; Bell et al., 2001; Clifford et al., 2011; Donyai et al., 2011; Mottram et al., 2002; Saade et al., 2018; Swallow et al., 2006; Wilbur, 2010). However, while there have been calls for PLT (Attewell et al., 2009; Donyai et al., 2011), relatively little research has examined how PLT could be delivered in practice and whether it indeed provides benefits to pharmacists and employers. This study contributes to addressing that gap by examining the feasibility of PLT provision as a mechanism for CPD access, and by proposing potential systemic, structural mechanisms for delivering PLT at scale: structured PLT slots and non-patient-facing hours. These models offer a foundation for further study, as well as potential policy and operational reform.

Participants’ support for large-scale PLT appears at odds with the low uptake of the programme (42 of approximately 1,000 community pharmacists applied). However, this could be explained to some degree by the reluctance that some pharmacists faced from their employers about pursuing the opportunity. Indeed, in 2023, 69% of UK pharmacists reported that they were expected to complete their CPD in their own time (Royal Pharmaceutical Society, 2023b), underscoring a culture of CPD being seen as an individual rather than an organisational responsibility. Given similar trends in other countries, it could be that such a culture is not unique to the UK.

Importantly, this study demonstrates that financial reimbursement alone is not a sufficient mechanism to overcome employer reluctance and structural barriers to PLT. Covering the *cost* of backfill does not extinguish the challenge of *sourcing* backfill, and could be an explanation for the six pharmacists who dropped out of the programme. Subsequently, sustainable PLT provision must go beyond funding to include workforce planning and operational redesign. Such insight is critical for stakeholders aiming to future-proof community pharmacy as pharmacists’ roles expand and services are increasingly pushed into community pharmacy from primary care (NHS, 2024).

In drawing comparisons across other pharmacy sectors, the findings echo international evidence showing that CPD tends to be more accessible in hospital and primary care settings (Alhaqan et al., 2021; Micallef & Kayyali, 2022). Without equivalent structures in community pharmacy, the concern around workforce attrition expressed by participants here is a real risk as pharmacists seek roles with more supportive learning environments (Smail, 2023).

This study does present limitations in terms of its scale and participant numbers. The context of high pressures faced by community pharmacy was not insignificant, and while the duration of interviews and the use of surveys may have limited the level of detail in data collection, if any more time had been demanded from participants, it would have risked lower participation. The pharmacists, being a self-selected group, may suggest that their utilisation of and support for PLT is not representative of the full community pharmacy workforce. However, these participants’ views align with the wider international literature. Methodologically, this study is strengthened by triangulating data collection tools (diaries, surveys and interviews) and sources (pharmacists, employers and E&T Leads). The constant comparative approach to analysis permitted ongoing review of participants’ perceptions, and comparisons across different stakeholders offers a broader perspective not limited to the single voice of the ‘consumer’ of PLT (pharmacists). Additionally, a realist approach facilitated a focus on delineating CMOs and particularly the mechanisms to address barriers to PLT and consolidate the benefits. Confirmability of interpretations and findings was supported through presentation of preliminary results to E&T Leads and their reflections.

## Conclusion

In a rapidly evolving pharmacy landscape, PLT is increasingly necessitated to support community pharmacist development, wellbeing, and the ability to deliver high-quality patient services. However, funding alone is insufficient to address the challenges faced by employers in providing PLT, and broader workforce planning and operational redesign are essential for sustaining PLT at scale.

This study confirms that PLT can produce positive outcomes not only for pharmacists, but also employers and patients. For pharmacists, PLT enhances confidence, scope of practice and wellbeing, particularly in contexts when their time is sufficiently backfilled and their workplace supports a learning culture. Employers can benefit through upskilled staff, increased services, and capacity to meet evolving healthcare demands of patients. Crucially, these outcomes are most reliably achieved in contexts where adequate staffing or locum availability prevents reduced productivity or financial consequences from releasing pharmacists.

Building on these findings, two promising models are posed for potential national implementation: pre-determined PLT slots, and regionally coordinated non-patient-facing hours. These models warrant further research and piloting to assess feasibility, acceptability, and impact at scale.

Regardless of model, in order to avoid fragmentation, workforce attrition and inadequate service quality, sustained investment and policy innovation are needed to ensure PLT is more widely integrated into community pharmacy practice. Without such changes, community pharmacy is vulnerable to growing service demand, workforce burnout, and declining patient dissatisfaction (Royal Pharmaceutical Society, 2023b; Smail, 2023).

## Supplementary Material

Supplementary Material.docx
