# Supplementary material for: Protected learning time in community pharmacy and possibilities for upscaling: an exploratory study in Wales, UK
Source: J Pharm Policy Pract. 2026 Feb 20;19(1):2629063. doi: 10.1080/20523211.2026.2629063 (PMC12927405; doi:10.1080/20523211.2026.2629063)
Supplement: Supplementary Material.docx [file JPPP_A_2629063_SM0273.docx]

**Supplemental Online Material**

**Evaluation of Models of Support for Community Pharmacy Registrants’ Development**

PARTICIPANT INFORMATION SHEET: Pharmacists

You are invited to take part in our evaluation of the new pilot models of support (protected time) for community pharmacy registrants’ development. Before you decide whether or not to take part, please read the following information carefully. If you have any questions, please contact [anonymised] whose contact details are provided at the end.

**What is the purpose of the research?**

The intention of this study is to evaluate the outcomes of the three new models of development support, providing evidence to inform future policy direction and the NHS Education Commissioning and Training Plan.

**Who is organising and funding this study?**

[anonymised]

**Why have I been invited to take part in the study?**

You have been invited to participate as you are a pharmacist enrolled on one of the three models of support.

**What will taking part involve?**

Taking part in the study will involve submitting a number of ‘online diaries’ relating to your use of the protected time, and participating in a focus group, and online survey.

Throughout the duration of the academic year, you will be invited to submit a short online ‘diary entry’ after each of your days of protected time. We will ask you to report on the activity you have undertaken during this protected time, any achievements you have obtained, the usefulness of the protected time and other general reflections. We will also ask you to provide your name, this is so that we can inform HEIW who has been using the protected time and backfill payments can be issued. It is important you understand our distinctly separate role from HEIW, whereby they are the programme providers, and we are the independent and external researchers. Us providing this information to them is to streamline the process. Your name will not be matched with the data you provide and the raw responses you provide will be confidential to the [anonymised] team and will not be shared with HEIW. No names or any other identifiable data will be extracted for analysis and you will not be identifiable in any report.

The focus group will take place approximately midway through the academic year and will involve you and other pharmacists on the same model of CPD support. Focus group participants will be asked about their view of any benefits of the model of support, how the protected time is being used and the value of support. You are not expected to share any information or opinion which you do not feel comfortable sharing. Should you provide permission freely, the focus group discussion will be audio recorded for later transcription. At this point, all data will be anonymised.

At the end of the academic year, you will be invited to complete an anonymous online survey in order to provide any final reflections around your experiences.

**Do I have to take part in the study?**

The only element of participation required from you is to use the online diaries. This is so that HEIW are aware of who has used their protected time and can issue the relevant backfill payments. However, you can be brief in your responses and can decline that your responses are used in the evaluation.

For the focus groups and online survey, your participation is voluntary. If you *do* decide to participate in the study, we will ask you to complete a consent form. You will be free to withdraw from participation in the study at any time, without giving reason but any data previously collected from you may be included in the study.

If you decide you *do not* wish to participate in the evaluation, you do not have to provide a reason and it will not impede your involvement in the model of CPD support, although you will be required to use the online diaries to indicate your uptake of the protected time so that we can inform HEIW who can be issued with the backfill payment.

**Will I be paid anything for taking part?**

No, there are no payments for taking part in this study.

**What are the possible benefits of taking part?**

Your participation in this study will involve sharing your views on the support model of protected time that you are undertaking. Although there are no direct benefits to you as a result of your participation, this information will be used to inform the future policy direction and the NHS Education Commissioning and Training Plan.

**What are the possible risks of taking part?**

The only foreseeable potential risk of participation in this study is some discomfort you may feel in sharing your views of the support model, and in the case of focus groups, sharing these views in the presence of other focus group participants. It is not our intension to cause discomfort and you are encouraged to only contribute opinions you feel comfortable sharing.

**Will my taking part in this study be kept confidential?**

Your online diary entries will require you to provide your name so that the research team can link together the diary entries from individuals and so that they can confirm with HEIW who has used the protected time. None of the raw content or responses will be shared with HEIW and will be confidential to the research team. Your name will not be extracted for analysis, and you will not be personally identifiable in any report.

All data provided by you and your fellow participants in the focus group will be anonymised on transcription and you will not be personally identifiable. However, you should understand the limits in confidentiality of focus group discussions in that any information you share will be known to other focus group participants. All focus group participants will be asked to respect the confidentiality of the discussion. All survey responses will be anonymous and diary entries confidential to the research team. If any identifiable information is provided, this will be anonymised and not included in report.

Data collected during the study will be kept strictly confidential and any personal information you provide will be managed in accordance with data protection legislation.

**What will happen to my personal data?**

The personally identifiable data collected from you and retained will be your diary entries which will include your name, and your consent form (should you provide it), which will include your name and signature. This information is collected so we know who has consented to participate in the study and so that we can log diaries entries across a range of participants who will be using their days of protected time at different points during the year. It will also enable us to better understand your responses in the wider context of other data we collect, and allow us to tailor any reminders we might issue. All other information provided by you will be anonymous.

Although this research study is funded by HEIW, no raw data will be shared with them, only the names of those who have utilised their protected time.

[Anonymised] is the Data Controller and is committed to respecting and protecting your personal data in accordance with your expectations and Data Protection legislation. The University Data Protection Officer can be contacted at [anonymised]

In providing data for this research, we will process it on the basis that it is part of our public task as a university established to advance knowledge and education through its teaching and research activities.

**What happens to the data at the end of the research project?**

Data will be retained in accordance with [anonymised] research ethics requirements and may be accessed by members of the research team and, where necessary, by members of the University’s governance and audit teams or by regulatory authorities. Anonymised data will be kept for a minimum of 5 years, or at least 2 years post-publication.

**What will happen to the results of the study?**

The principal output will be a report documenting the evaluation of the three different models of support for CPD. This report will be shared with HEIW, however they will not have access to your personal data and will only see the anonymised report. It is expected that this report will be available in September 2022, although this is subject to change.

We may also report the results in academic journals and at relevant conferences. All data will remain anonymous, and participants will not be personally identified in any report, publication or presentation.

**What if there is a problem?**

The lead for this evaluation, [anonymised] will be available to answer any questions or queries regarding any aspects of the study. If you wish to complain or have concerns about the way you have been approached or treated during the course of this study, please contact the research ethics committee at [anonymised].

**Who has reviewed this study?**

This study has been reviewed and given a favourable opinion by the School of Social Sciences’ Research Ethics Committee at [anonymised]

**Further information and contact details**

[anonymised]

**Thank you for considering participation in this study.**

**Evaluation of Models of Support for Community Pharmacy Registrants’ Development**

PARTICIPANT INFORMATION SHEET: E&T Leads

You are invited to take part in our evaluation of the new pilot models of support for community pharmacy registrants’ development. Before you decide whether or not to take part, please read the following information carefully. If you have any questions, please contact [anonymised] whose contact details are provided at the end.

**What is the purpose of the research?**

The intention of this study is to evaluate the outcomes of the three new models of development support, providing evidence to inform future policy direction and the NHS Education Commissioning and Training Plan.

**Who is organising and funding this study?**

[anonymised]

**Why have I been invited to take part in the study?**

You have been invited to participate as you are a Pharmacy Lead at HEIW and involved in the Education and Training of pharmacists in Wales.

**Do I have to take part in the study?**

No, your participation is entirely voluntary. If you *do* decide to participate in the study, we will ask you to complete a consent form. You will be free to withdraw from participation in the study at any time, without giving reason but any anonymous data previously collected from you may be included in the study.

If you decide you *do not* wish to participate, you do not have to provide a reason and it will not impede your involvement in the support model.

**What will taking part involve?**

Taking part in the study will involve participating in a focus group at the end of the academic year (end of the pilot of support models). Yourself and other pharmacy leads at HEIW will be invited to participate in the focus group. Focus group participants will be asked about your views on the benefits of the individual support models, how pharmacists used the time, and what the future of support models might look like. You are not expected to share any information or opinion which you do not feel comfortable sharing. Should you provide permission freely, the focus group discussion will be audio recorded for later transcription. At this point, all data will be anonymised.

**Will I be paid anything for taking part?**

No, there are no payments for taking part in this study.

**What are the possible benefits of taking part?**

Your participation in this study will involve sharing your views on the three support models of protected time currently offered by HEIW. Although there are no direct benefits to you as a result of your participation, this information will be used to inform the future policy direction and the NHS Education Commissioning and Training Plan.

**What are the possible risks of taking part?**

The only foreseeable potential risk of participation in this study is some discomfort you may feel in sharing your views of the support models in the presence of other focus group participants. It is not our intent to cause discomfort and you are encouraged to only contribute opinions you feel comfortable sharing.

**Will my taking part in this study be kept confidential?**

All data provided by you and your fellow participants in the focus group will be anonymised on transcription and you will not be personally identifiable. However, you should understand the limits in confidentiality of focus group discussions in that any information you share will be known to other focus group participants. All focus group participants will be asked to respect the confidentiality of the discussion. Data collected during the study will be kept strictly confidential and any personal information you provide will be managed in accordance with data protection legislation.

**What will happen to my personal data?**

The only personally identifiable data collected from you and retained will be your consent form (should you provide it), which will include your name and signature. This information is only collected so we know who has consented to participate in the study. All information provided by you will be anonymous and will not be matched to the information in your consent form. Your consent form will be retained in accordance with [anonymised] research ethics requirements and may be accessed by members of the research team and, where necessary, by members of the University’s governance and audit teams or by regulatory authorities. Anonymised data will be kept for a minimum of 5 years, or at least 2 years post-publication. Although this research study is funded by HEIW, no raw data will be shared with them.

[anonymised] is the Data Controller and is committed to respecting and protecting your personal data in accordance with your expectations and Data Protection legislation. The University Data Protection Officer can be contacted at [anonymised]

In providing data for this research, we will process it on the basis that it is part of our public task as a university established to advance knowledge and education through its teaching and research activities.

**What will happen to the results of the study?**

The principal output will be a report documenting the evaluation of the three different models of support. This report will be shared with HEIW, however they will not have access to your personal data and will only see the anonymised report. It is expected that this report will be available in late 2022, although this is subject to change.

We may also report the results in academic journals and at relevant conferences. All data will remain anonymous, and participants will not be personally identified in any report, publication or presentation.

**What if there is a problem?**

The lead for this evaluation, [anonymised] will be available to answer any questions or queries regarding any aspects of the study. If you wish to complain or have concerns about the way you have been approached or treated during the course of this study, please contact the research ethics committee at [anonymised].

**Who has reviewed this study?**

This study has been reviewed and given a favourable opinion by the School of Social Sciences’ Research Ethics Committee at [anonymised].

**Further information and contact details**

[anonymised]

**Thank you for considering participation in this study.**

**Invitation email to pharmacists**

*Email Subject Head: Invitation to Feedback Focus Group – Pharmacy Protected Time*

Dear [name],

**You are invited to participate in a focus group discussion to feedback and discuss your experiences of the CPD model for Protected Learning Time.**

The focus group is an opportunity for you and others on this model to provide feedback on your experiences, its value as well as any challenges you may have faced. **You may use your protected time allocation to attend this event.**

The focus group will be conducted by [anonymised] as part of our evaluation of HEIW’s three models of support.

The focus group will take place on **Microsoft Teams,** and we are offering two timeslots:

**Monday 21^st^ March, 11:00 – 12:00**

**Monday 21^st^ March, 12:00 – 13:00**

**Monday 21^st^ March, 13:00 – 14:00**

**Monday 21^st^ March, 14:30 – 15:30**

**Tuesday 22^nd^ March, 11:00 – 12:00**

**Tuesday 22^nd^ March, 14:30 – 15:30**

**If you are able to participate in a focus group at one of these time slots, please contact [anonymised] directly on [anonymised email address].** If you cannot attend either of these timeslots, please contact us and we will strive to find an alternative solution.

An Information Sheet is attached to this email with further details on the study, what participation involves and how data will be used and stored.

Please do not hesitate to get in touch if you have any queries.

Many thanks and best wishes,

[anonymised]

**Pharmacist Online Diary (example)**

**
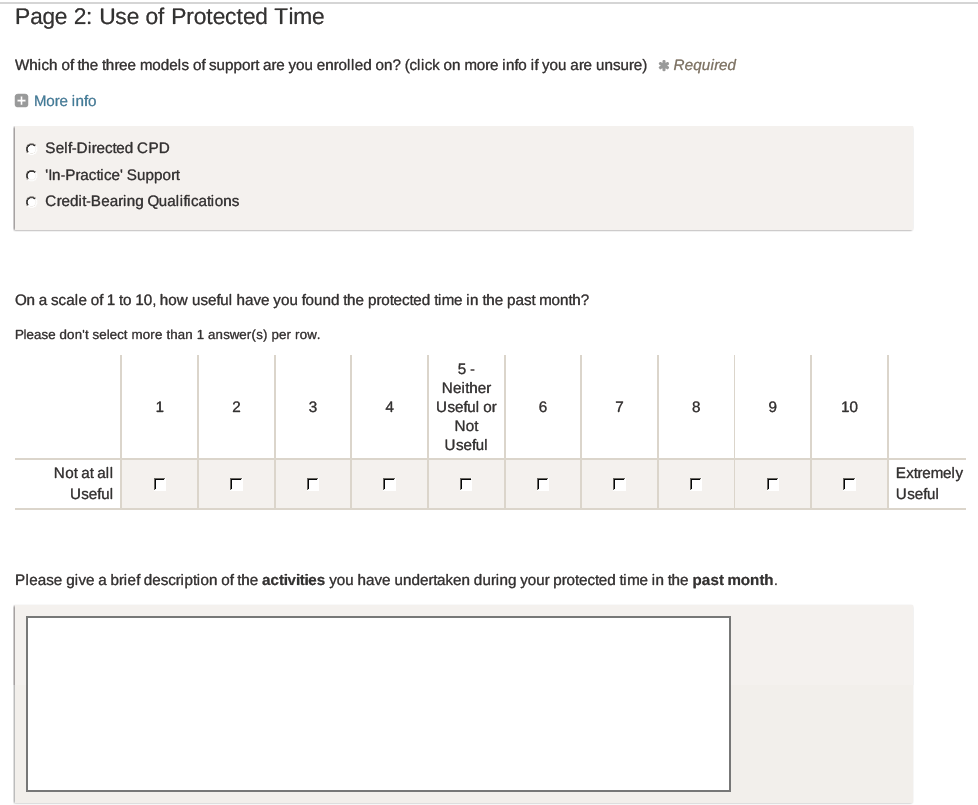
**

**
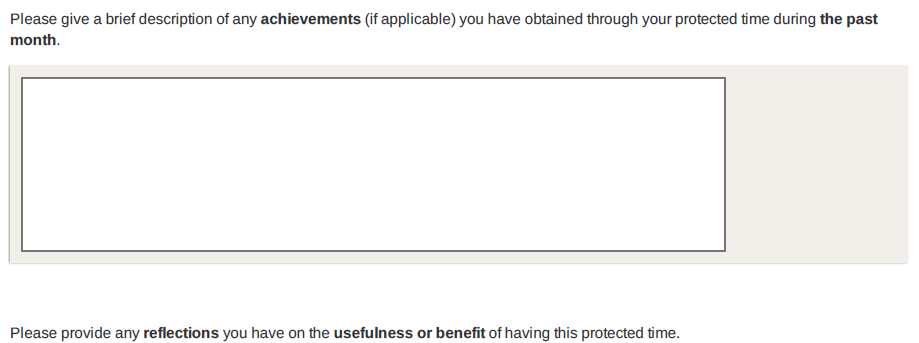
**

**
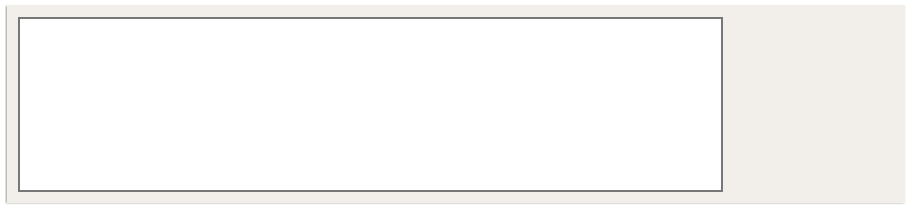
**

**Pharmacist Interview Question Schedule**

**Question Schedule: Self-directed CPD**

Motivations

Taking part in the protected time for CPD initiative was optional. What motivated you to take up the offer?

Were your **employers supportive** of your decision?

What were you hoping to **gain** from this model of protected time?

**What came first** – did you find out about the opportunity for protected time and then looked for a relevant learning framework, or did you want to pursue a particular framework and then saw the protected time programme as a vehicle to do so?

Arranging Time

How have you found the process of **arranging** your protected time?

Have your managers been **supportive**?

How easy has it been to **align** your booked time with when you need to take it?

Have there been any additional **pressures** due to workforce shortages?

Use of Time

How have you typically been **using** the protected time? What **activities** do you undertake?

How have you found the **self-directed** element and need to **plan your own learning**?

Have you engaged with a mentor during your PLT?

If yes - What is the **nature of support** that your mentor provides?

If no – Do you think there would be any **added value of having a mentor**?

Have you used the time to pursue any particular credit-bearing modules or qualifications?

If yes - What **module(s)** are you pursuing with your protected time?

**Why** did you choose that particular module(s)?

Is your module(s) linked to an **assessment**? Do you expect to **fulfil the award** through your protected time? (if not, why not?)

Impact of Time

How have you found having the opportunity to **step away from your patient-facing role**?

So far, what have you **gained** from having the protected time?

Prompt: particular achievements or learning gains? Qualifications?

Prompt: soft skills, e.g. time management, confidence, leadership

Do you feel your use of the protected time has had an **impact on your practice**?

Benefits, Challenges, Improvements

What do you see as the biggest **benefit** of pursuing the self-directed CPD model?

Do you feel the protected time has had an impact on your levels of **job satisfaction** or **wellbeing**?

Have there been any **challenges** from pursuing this model?

What is your view on **how HEIW provided** the protected learning time?

Do you have any suggestions for **improvements**?

Do you have any other comments?

**Employer Survey**


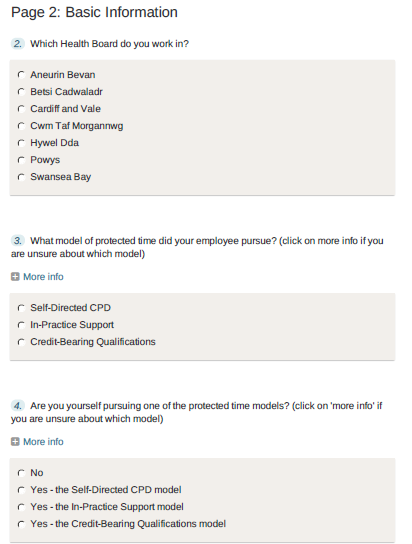


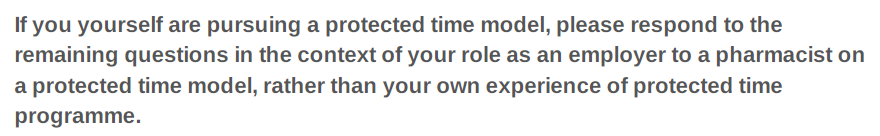


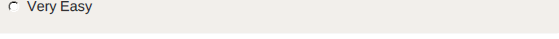

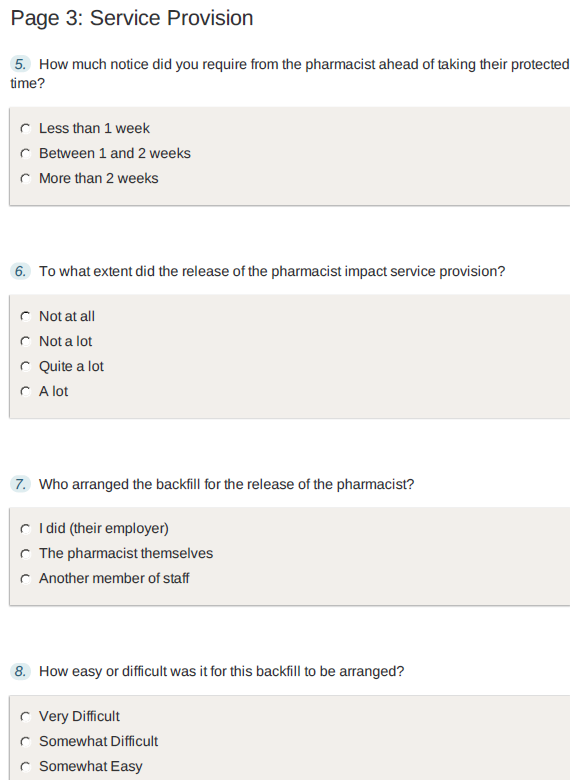


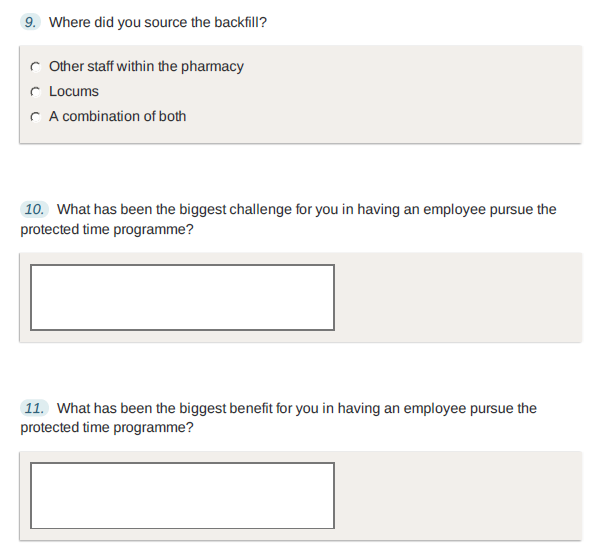


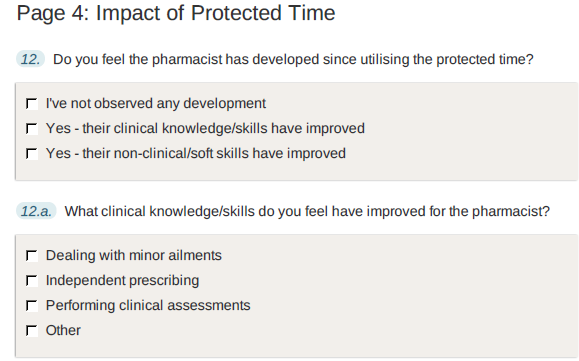


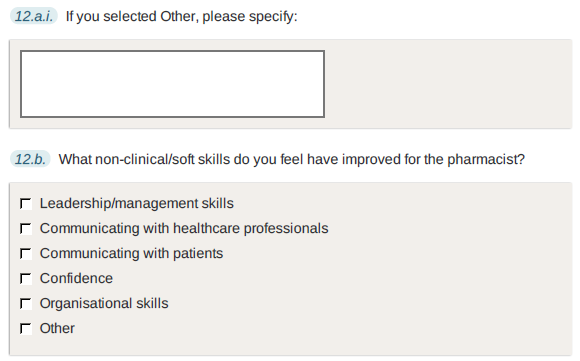


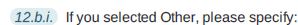


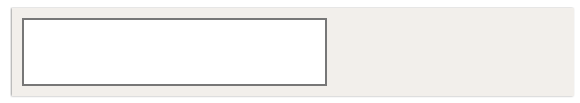


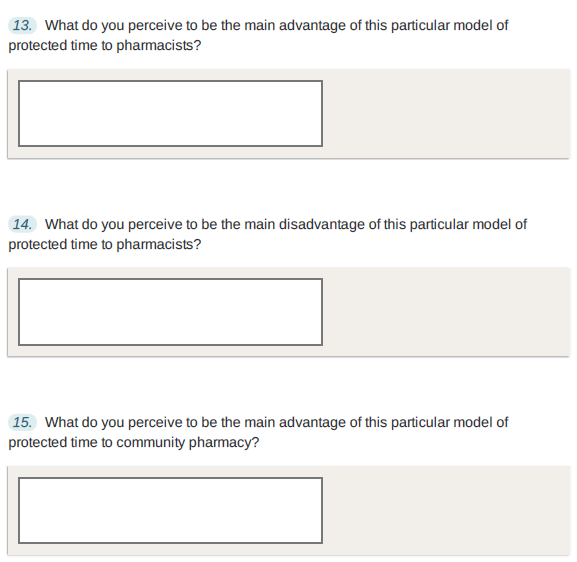


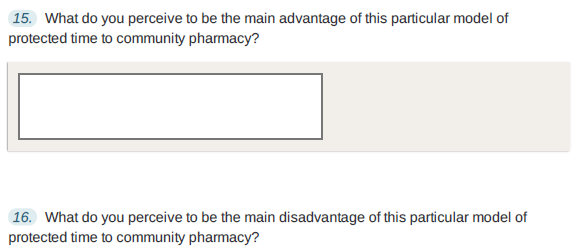


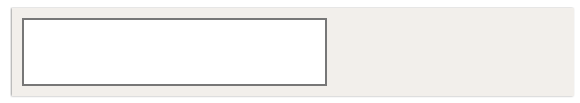


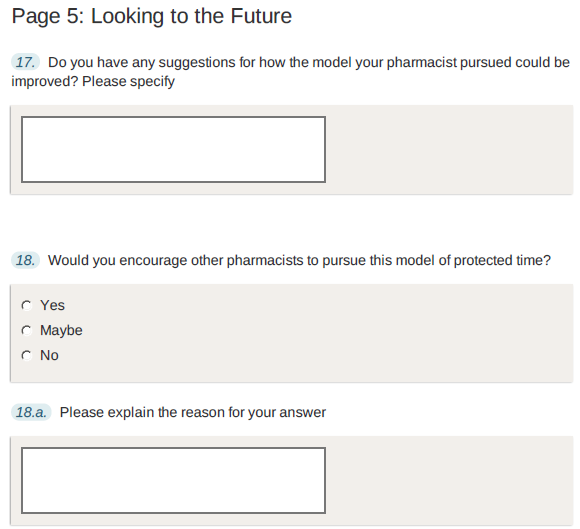


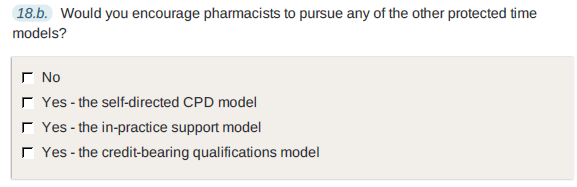


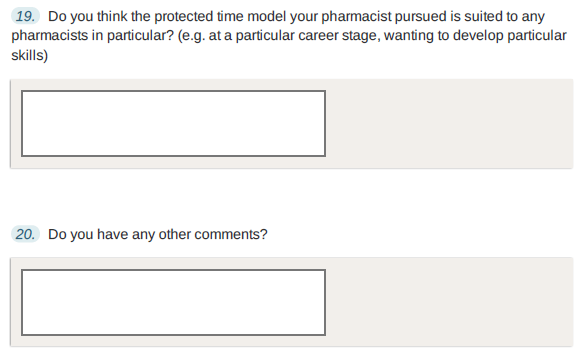


**Education and Training Leads Interview Question Schedule**

**Questions**

Begin by inviting general feedback and commentary on the results presented.

**General experiences**

What have been your experiences within your role on the pilot?

- What engagement have you had with pharmacists on the models?
- What engagement have you had with employers?
- What engagement have you had with mentors on the in-practice support model?

What challenges have you faced?

How were they overcome?

**Different models**

What have been your experiences of the different models?

- Any more difficult/easier to manage?
- How do the outputs of different models compare? (note: in-practice support most expensive)

What are your reflections on pharmacist’s uncertainty on which model they were pursuing?

What do you perceive as the best way of guaranteeing time is accounted for? (e.g. diaries, qualification, evidence portfolio)

What are your views on the return on investment for this scheme?

- Value for money?

**Looking to the future**

What do you see protected time looking like in the future? All three models? One model?

Views on upscaling – how they feel this might be managed?

- Accounting for increased demand
- Financial implications
- Ensuring backfill available
- Impact on service provision
- Comparison with GP model – closing for an afternoon

Any other comments?

Thank you for your participation.
